# The PI3K pathway as a therapeutic intervention point in inflammatory bowel disease

**DOI:** 10.1002/iid3.435

**Published:** 2021-05-04

**Authors:** Paula Winkelmann, Anna‐Lena Unterweger, Diya Khullar, Florian Beigel, Leandra Koletzko, Matthias Siebeck, Roswitha Gropp

**Affiliations:** ^1^ Department of General, Visceral, and Transplantation Surgery University Hospital, LMU Munich Germany; ^2^ Department of Medicine II University Hospital, LMU München Germany; ^3^ Present address: 41 Drum Hill Drive, Summit, NJ 07901 USA

**Keywords:** copanlisib, immune‐metabolism, inflammatory bowel disease, NSG‐UC mouse model, PI3K, ulcerative colitis

## Abstract

With glucose being the preferred source of energy in activated T cells, targeting glycolysis has become an attractive therapeutic intervention point for chronic inflammatory bowel diseases (IBD). The switch to glycolysis is mediated by phosphoinositide‐3‐kinases (PI3K) which relay signals from surface receptors to the AKT pathway. We first confirmed by analysis of the oxygen consumption rate (OCR) and extracellular acidification rate (ECAR) that metabolism is shifted towards glycolysis in IBD patients as compared to non‐IBD donors. In contrast to non‐IBD donors, OCR correlated with ECAR (IBD: cor = 0.79, *p* = 2E‐10; non‐IBD: cor = 0.37, *p* = n.s.), in IBD patients. Second, we tested the PI3K inhibitor copanlisib as a potential therapeutic. Ex vivo, copanlisib suppressed the ECAR significantly in T cells activated by anti‐CD3 antibodies and significantly decreased ECAR rates in the presence of copanlisib (anti‐CD3: 58.24 ± 29.06; copanlisib: 43.16 ± 20.23, *p* < .000. In addition, copanlisib impaired the activation of CD4^+^ CD25^+^ T cells (anti‐CD3: 42.15 ± 21.46; anti‐CD3 + copanlisib: 26.06 ± 21.82 *p* = .013) and the secretion of cytokines (IFNγ: anti‐CD3: 6332.0 ± 5707.61 pmol/ml; anti‐CD3 + copanlisib: 6332.0 ± 5707.61, *p* = .018). In vivo, copanlisib significantly improved the histological scores (ethanol: 8.5 ± 3.81; copanlisib: 4.57 ± 2.82, *p* = .006) in the NSG‐UC mouse model. Orthogonal partial least square analysis confirmed the efficacy of copanlisib. These data suggest that the PI3K pathway provides an attractive therapeutic intervention point in IBD for patients in relapse. Targeting metabolic pathways have the potential to develop phase dependent therapies.

## Graphical abstract

In response to an inflammatory assault T cells favor glycolysis to ensure swit energy supply required for proliferation, migration and secretion of cytokines, chemokines and growth factors. Therefore, targetinginflammation has come into focus of interest as a therapeutical intervention point in chronic inflammatory bowel diseases. The phosphoinositide‐3‐kinases inhibitor copanlisib inhibits glycolysis and suppresses inflammation in a mouse model of ulcerative colitis.

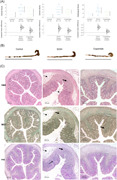

**Abbreviations**
Akt: proteinkinasen B (Akt1‐3)
FELASA: Federation of Laboratory Animal Science Association
HE: hematoxylin and eosin
IFNγ: interferonγ
IL: interleukin
LPMC: lamina propria mononuclear cells
MGT: Masson Goldner Trichrome
NSG: NOD/scid IL‐2Rγ^null^
oPLS‐DA: orthogonal partial least square discrimination analysis
PAS: periodic acid Schiff
PBMC: peripheral blood mononuclear cells
PBS: phosphate buffered saline
PCA: principal component analysis
PI3K: phosphoinositid‐3‐Kinase
RMSEE: root mean square error of estimation
SCCAI: Simple clincal colitis activity index
UC: ulcerative colitis

## 1 INTRODUCTION

colitis (UC) and Crohn's disease (CD) belong to the chronic inflammatory bowel disease (IBD) of unknown etiology. Although both diseases share common clinical features such as abdominal pain, diarrhoea, weight loss, and fatigue, they represent two distinct forms of chronic inflammation of the gastrointestinal tract and, as such, have different causes and different pathogenic mechanisms.^1^, ^2^ Unlike CD, which can cause inflammation in any part of the digestive system and is a transmural and stricturing disease, UC solely affects the mucosa and is restricted to the colon.^3^, ^4^, ^5^ Although the paradigm has fallen that described CD as a TH1,^6^, ^7^ and UC as TH2 driven disease,^8^, ^9^ and it has been shown that monocytes also drive inflammation in IBD,^10^, ^11^, ^12^ activation of TH cells to include TH17 cells remains an important therapeutic intervention point for both diseases. Therefore, calcineurin inhibitors tacrolimus and cyclosporine are often used to suppress the synthesis of inflammatory cytokines by T cells. This also occurs in combination with biologicals when monotherapies fail to achieve remission. In recent years, however, insights into immuno‐metabolism have opened up new routes of therapeutic intervention. Activation of T cells is accompanied by a metabolic switch to glycolysis which ensures swift energy supply and provides biosynthetic intermediates required for proliferation, migration, differentiation and synthesis of interleukins (ILs), chemokines and growth factors.^13^, ^14^ This metabolic switch has been observed in patients with UC and CD in relapse as compared to patients in remission and to nondiseased individuals. Metabolic profiling identified glycolysis as the preferred metabolic pathway in acute phases of disease^15^, ^16^; hence, suppressing glycolysis might open up entirely novel treatment options for UC or CD.^17^ In this setting, phosphoinositide‐3‐kinases (PI3Ks) have come into focus of interest as they act as a central node to relay signals from surface receptors to downstream mediators. Surface receptors include antigen‐T cell receptor (TCR) complexes as well as IL receptors and growth factor receptors among others. PI3Ks catalyse the conversion of phosphatidylinositol‐4,5‐bisphosphate to phosphatidylinositol‐3,4,5‐trisphosphate which, in turn, activates Ras homologue (RHO) GTPases and the NFκB‐, SH1P‐ and AKT pathways. The AKT pathway plays a central role in immune‐metabolism as it controls glycolytic enzymes and expression levels of the glucose transporter GLUT.^18^ Therefore, PI3K seems a promising therapeutic target in inflammation. As the metabolic needs of malignant cells are similar to those of immune cells, PI3K inhibitors have first been developed for leukaemia. Copanlisib is an example of a pan‐class I phosphoinositide 3‐kinase inhibitor with particular activity against the α and δ isoforms^19^ and is now approved for treatment of follicular lymphoma. Since mainly PI3K‐δ mediates TCR and cytokine receptor downstream signalling to promote inflammation and cell survival, we assumed that the PI3K‐δ inhibitor copanlisib maybe especially suited to suppress inflammation.^20^, ^21^, ^22^

Mouse animal models for IBD are important tools to validate novel therapeutics. Unfortunately, they poorly reflect the human disease, and results obtained in these models may not be translated to clinical trials. To narrow the gap between preclinical and clinical studies, we have developed a mouse model that is based on NOD/scid IL‐2Rγ^null^ mice (NSG) reconstituted with peripheral blood mononuclear cells (PBMCs) from human patients suffering from UC (NSG‐UC). In this model, colitis‐like symptoms are induced by rectal application of ethanol and pathological manifestations include changes in crypt architecture, influx of inflammatory cells, edema, crypt loss, goblet cell loss and fibrosis. This model reflects the disease background of the donor and allows for addressing human molecular targets expressed in leukocytes.^23^

In this study, we examined whether suppressing glycolysis by PI3K inhibition offers a treatment option in IBD. First, we analysed the T cell activation and the metabolic status in IBD patients as compared to non‐IBD donors. PBMCs were subjected to flow cytometric and oxygen consumption rate (OCR) and extracellular acidification rate (ECAR) analysis and frequencies of early activated CD4^+^ CD69^+^ and correlation of oxygen consumption and lactate synthesis were measured. The inhibitory capacity of copanlisib was examined ex vivo and in vivo in the NSG‐UC mouse model. Data presented here indicate that the activated inflammatory status was accompanied by increased glycolysis. Ex vivo, copanlisib significantly inhibited T cell activation and cytokine secretion. In vivo, copanlisib ameliorated the pathological phenotype in NSG mice and suppressed the activation of CD4^+^ CD69^+^ cells.

## 2 MATERIALS AND METHODS

### 2.1 Isolation of PBMC

A total of 20–60 ml of peripheral blood in trisodium citrate solution (S‐Monovette; Sarstedt) were collected from the arm vein of UC and CD patients as described previously.^10^ The blood was diluted with Hank's balanced salt solution (Sigma Aldrich) in a 1:2 ratio and lloaded onto LeucoSep tubes (Greiner Bio One). Tubes were centrifuged at 400*g* for 30 min without acceleration and break and PBMC were extracted from the interphase and diluted with phosphate buffered saline (PBS) to a final volume of 40 ml. Cells were counted and centrifuged at 1400*g* for 5 min. The cell pellet was resuspended in PBS at a concentration of 4 × 10^6^ cells in 100 µl.

### 2.2 Flow cytometric analysis

All antibodies (Table S1) were purchased and used according to the manufacturer's instructions (Biolegend). Flow cytometry was performed using a Thermofisher Attune NxT (Thermo Fisher Scientific) and analysed with FlowJo 10.1‐Software (FlowJo LLC).

### 2.3 Seahorse analysis

PBMCs were isolated from 10 ml of blood and seeded into the Agilent Seahorse XFp Cell Culture Miniplates (Agilent Technologies Inc.) to a density of 250000 cells/well. The assay medium consisted of Roswell Park Memorial Institute (RPMI) Medium (Thermo Fisher Scientific) RMPI, 100 nM pyruvate, 100 nM glutamine, and 100 g/L glucose (Agilent Technologies Inc.). Postseeding, the cells were kept at 37°C for 1 h, before the assay was performed. Patient samples were measured in triplicates or quintuplicates in the ground state without further activation. To measure the impact of copanlisib, cells were pre‐incubated with anti‐CD3 (61 ng/ml; BioLegend) for 1 h at 37°C in the presence or absence of copanlisib (100 nM/ml). Energy state was measured in the ground state for 1 h at 10 time points. For measurements, a Seahorse XFp Extracellular Flux Analyzer (Agilent Technologies Inc.) was used.

### 2.4 Cell culture

PBMCs from healthy donors were isolated from 10 ml blood. The cell pellet was resuspended in RPMI Medium (Thermo Fisher Scientific) at a concentration of 1 × 10^6^ cells/ml as described previously.^24^ Cells were incubated with additional 500 μl RPMI with 10% fetal calf serum (FCS) and 1% penicillin‐streptomycin (Sigma‐Aldrich). Wells incubated in the absence or presence of anti‐CD3 (61 ng/ml; BioLegend) served as negative and positive controls, respectively. Copanlisib was added at a concentration of 100 nM.

### 2.5 Study protocol

Mice were obtained from Charles River Laboratories and kept under specific pathogen‐free conditions in individually ventilated cages in a facility controlled according to the Federation of Laboratory Animal Science Association guidelines as described previously.^25^ Six‐ to eight‐week old NOD.cg‐Prkdc^SCID^ Il2rg^tm1Wjl^/Szj mice (abbreviated as NOD‐scid IL‐2Rγ^null^, NSG) were engrafted with 100 µl PBMC cell solution (4 × 10^6^) into the tail vein on Day 1 as previously described^25^ and presensitized by rectal application of 150 µl 10% ethanol on Day 7 using a 1 mm catheter (Henry Schein). The catheter was lubricated with lidocaine 2% gel (AstraZeneca).^25^ Rectal application was performed under general anesthesia using 4% isoflurane. Following application mice were kept at an angle of 30° to avoid ethanol dripping. On Day 14 mice were additionally challenged with 50% ethanol following the protocol of Day 8. Copanlisib was applied intraperitoneally (i.p) at a concentration of 6 mg/kg in 0.5% methylcellulose gel in PBS (Firma Cat# M0512; Merck KGaA) on Days 7, 8, 14, 15, and 16. Mice were sacrificed on Day 18.

### 2.6 Clinical activity score

The assessment of severity of colitis was performed daily as previously described^26^: Loss of body weight: 0% (0), 0%–5% (1), 5%–10% (2), 10%–15% (3), 15%–20% (4). Stool consistency: formed pellet (0), loose stool or unformed pellet (2), liquid stools (4). Behaviour: normal (0), reduced activity (1), apathy (4) and ruffled fur (1). Body posture: Intermediately hunched posture (1), permanently hunched posture (2). The scores were added daily into a total score with a maximum of 15 points per day. Animals who suffered from weight loss more than 20%, rectal bleeding, rectal prolapse, self‐isolation or a severity score more than 7 were euthanized immediately and not taken into count. All scores were added for statistical analysis.

### 2.7 Macroscopic colon score

The colon was removed, and the colon was scored.^26^ Pellet: formed (0), soft (1), liquid (2); length of colon: more than 10 cm (0), 8–10 cm (1), less than 8 cm (2); Dilation: no (0), minor (1), severe (2); Hyperemia: no (0), yes (2); Necrosis: no (0), yes (2).

### 2.8 Histopathology

Sections from distal parts of the colon were fixed in 4% formaldehyde for 24 h, stored in 70% ethanol and embedded in paraffin as described previously.^25^ A total of 3 µm sections were cut and stained with haematoxylin and eosin (HE), periodic acid‐Schiff (PAS) and Masson‐Goldner trichrome (MGT, all from Morphisto GmbH).

Epithelial erosions were scored as described prviously^26^: no lesions (1), focal lesions (2), multifocal lesions (3). Inflammation was scored as follows: infiltration of few inflammatory cells into the lamina propria (1), major infiltration of inflammatory cells into the lamina propria (2), confluent infiltration of inflammatory cells into the lamina propria (3), infiltration of inflammatory cells including tunica muscularis (4). Fibrosis was scored as follows: focal fibrosis (1), multifocal fibrosis and crypt atrophy (2) and general fibrosis and crypt atrophy (3). The presence of edema was scored as follows: focal (1), multifocal (2), general (3). Hyperemia was scored with one additional point. The scores for each criterion were added into a total score. Images were taken with an AxioVert 40 CFL camera (Zeiss) using the Zeiss ZE n2 lite software. Representative longitudinal sections in original magnification are shown. To enhance contrast of the pictures, Adobe Photoshop CC a tonal correction was used.

### 2.9 Isolation of human leucocytes

Spleens were minced and cells filtrated through a 70 µl cell strainer followed by centrifugation at 1400*g* for 5 min and suspended in FACS buffer (1X PBS, 2 mM ethylenediaminetetraacetic acid, 2% FCS) to isolate human leukocytes as previously described.^26^ Cell suspensions were additionally filtrated using a 35 µm cell strainer and then labelled for flow cytometry analysis.

### 2.10 Statistical analysis

Statistical analysis was performed with R: A language and environment for statistical computing (R Foundation for Statistical Computing, Vienna, Austria; URL https://www.R-project.org/). Variables in the supplementary were represented with mean, *SD*, N, difference and 95% confidence interval (CI). A two‐sided Student's *t* test and a CI of 95 was used to compare binary groups and for more than two groups, analysis of variance (ANOVA) followed by the post hoc Tukey's honestly significant difference (TukeyHSD) test was conducted. Variables subjected to ANOVA were tested for normal distribution. All variables fulfilled this requirement. Cummingplots were generated using the dabestr package and are used in this study for data presentation and comparison. Cumming plots are a new generation of data analysis with bootstrap‐coupled estimation plots that move beyond *p* values.^27^ These plots are used to analyze large samples and multiple groups. The estimation graphics mainly offer five key advantages: (1) The plot of the full sampling‐error curve of the effect size presents the distribution's graded nature, (2) the difference axis increases transparency to the comparison being made and (3) in comparison to *p* values, which conflate the magnitude and precision in a single number, the relative size of the CI provides a specific measure of its precision.^27^ (4) The sampling‐error curve is derived with bootstrapping, which makes the method robust and versatile and (5) the difference diagram encourages the quantitative reasoning about the system under study by focusing on an effect size.^27^ Orthogonal partial least square discrimination analysis (oPLS‐DA) was performed using the ropls package.

## 3 RESULTS

### 3.1 Ex vivo analysis of IBD and non‐IBD donors revealed differences in T cell activation and metabolism

**Figure 1:**
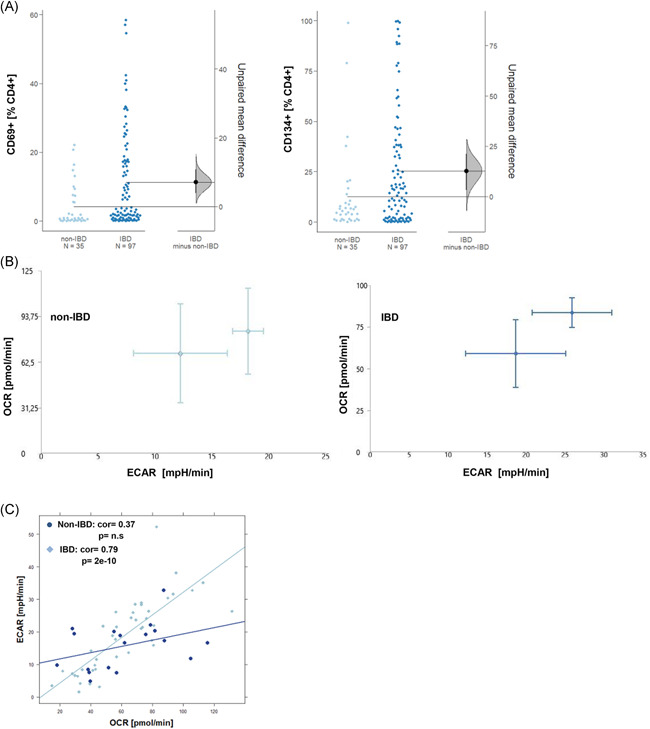
Activation status of T cells and metabolic activity is affected in IBD patients. (A) Flow cytometric analysis of early‐activated (CD4^+^ CD69^+^) and late‐activated (CD4^+^ CD134^+^) in PBMCs from IBD (number of donors *N* = 77) and non‐IBD (number of donors *N* = 36). Frequencies are depicted as Cumming plots. The upper part of the plot presents each data point in a swarm plot. The mean and *SD* of each group is plotted as a gapped line, where the vertical lines correspond to the mean ± *SD* and the mean itself is depicted as a gap in the line. In the lower panel of the plots, the effect sizes are shown. The 0 point of the difference axis is based on the mean of the reference group (control). The dots show the difference between groups (effect size/mean difference). The shaded curve shows the entire distribution of excepted sampling error for the difference between the means (the higher the peak, the smaller the error). The error bar in the filled circles indicates the 95% confidence interval (bootstrapped) for the difference between means. (B) Representative energy maps of IBD patients and non‐IBD patients determined by Seahorse technology. (C) Pearson's product‐moment correlation analysis of ECAR and OCR rates (IBD: number of donors *N* = 10, experiments were performed in triplicates or quintuplicate, number of samples *n* = 42; non‐IBD: number of donors *N* = 6, number of samples *n* = 18). Cor, correlation coefficient; ECAR, extracellular acidification rate; OCR, oxygen consumption rate

To analyze the activation status of T cells, PBMCs from IBD patients (*N* = 97) patients and non‐IBD donors (*N* = 35) were isolated and subjected to flow cytometric analysis as described in Section 2. Frequencies of CD4^+^ T cells expressing the early activation marker CD69^+^ or the late activation marker CD134^+^ (Ox40) were determined (For basic patient demographics see Table 1, for gating strategy see Figure S1). Data are presented as Cumming plots.^28^ As shown in Figure 1A, frequencies of both cell types were highly variable in all groups reflecting the dynamics of inflammatory processes.^29^ As indicated by nonoverlapping CI of the unpaired mean differences frequencies both cell types were significantly different in the IBD group as compared to non‐IBD group (CD4^+^ CD69: *p* = 1E‐04; CD4^+^ CD134: *p* = 7E‐03; Welch two‐sample *t* test). To evaluate whether the activation status was reflected in cell metabolism, PBMCs of IBD patients (*n* = 10) and non‐IBD donors (*n* = 6) were subjected to OCR and ECAR analysis as described in Section 2 (For OCR and ECAR mean values and donor characteristic see Table S2). In this assay, oxidative phosphorylation is determined by measuring the OCR and glycolysis by measuring the ECAR caused by lactate as a byproduct of glycolysis. As shown in Figure 1B, The energy map of two different IBD patients in the ground state suggested increased glycolytic activity as indicated by slightly higher ECAR values as compared to the two different non‐IBD donors. No correlation of the OCR or the ECAR was observed with age or body mass index. As the variability of ECAR and OCR was high in both groups, the correlation of the ECAR and OCR rates was analyzed by Pearson's product‐moment correlation analysis. In contrast to the non‐IBD group, OCR and ECAR were significantly correlated in the IBD group suggesting that in this group, oxygen consumption was coupled to glycolysis (non‐IBD: cor = 0.37, *p* = .12, 95% CI = −0.11–0.71 IBD: cor = 0.79, *p* = 2e‐10, 95% CI = 0.65–0.88).

**Table 1:** Basic patient demographics

	UC	CD	Non‐IBD
	*N* =	*N* = 27	
Age (years)			
Mean (*SD*)	37.81 (14.31)	46.6 (17.7)	38.0 (16.65)
Range	23–80	21–74	21–66
Gender (% male)	55	55	38
Duration of UC/CD (years)			
Mean (*SD*)	11.14 (8.62)	17.2 (11.33)	
Range	1–39	2–41	
SCCAI^30^/CDAI^31^			
Mean (*SD*)	4.48 (3.19)	52.07 (50.68)	
Range	0–12	21–200	
Treatment (current)			
TNF‐α‐blocker	28	13	
Vedolizumab	20	5	
Ustekinumab		1	
Mesalazine	18	2	
Glucocorticoids	4	3	
Azathioprine	4	3	

Abbreviations: CD, Crohn's disease; TNF, tumor necrosis factor; UC, Ulcerative colitis.

### 3.2 Copanlisib suppresses glycolysis in activated T cells

**Figure 2:**
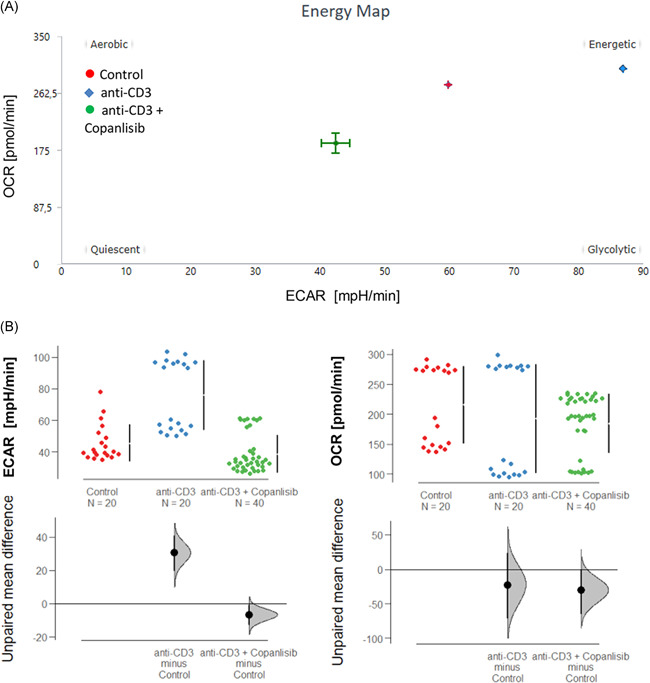
Copanlisib affects glycolysis in activated CD4^+^ T cells. OCR and ECAR analysis of PBMCs performed with two different donors (*N* = 2) incubated for 2h in the presence or absence anti‐CD3 monoclonal antibodies (61 ng/ml) and copanlisib (100 nM). Measurements were taken over the course of 1 h at 10 consecutive time points. The copanlisib group was measured in duplicates. Number of measurements (control: *n* = 20; anti‐CD3: *n* = 20; copanlisib: *n* = 40) Groups compared: untreated control (control), activated CD4^+^ T cells (anti‐CD3) and activated CD4^+^ T cells + copanlisib (anti‐CD3 + copanlisib). (A) Representative energy map of control‐, anti‐CD3, and anti‐CD3 + copanlisib groups. (B) Measurements of consecutive time points depicted as Cumming plots. The upper part of the plot presents each data point in a swarm plot. The mean and *SD* of each group is plotted as a gapped line, where the vertical lines correspond to the mean ± *SD* and the mean itself is depicted as a gap in the line. The 0 point of the difference axis is based on the mean of the reference group (control). The dots show the difference between groups (effect size/mean difference). The shaded curve shows the entire distribution of excepted sampling error for the difference between the means (the higher the peak, the smaller the error). The error bar in the filled circles indicates the 95% confidence interval (bootstrapped) for the difference between means. ECAR, extracellular acidification rate; OCR, oxygen consumption rate; PBMC, peripheral blood mononuclear cell

As copanlisib exerts its activity on the AKT pathway, we would expect that the inhibitory activity of copanlisib is reflected in cell metabolism. To prove this hypothesis, the energy consumption of PBMCs was analyzed by Seahorse technology as described in Section 2. PBMCs were seeded into the wells at a concentration of 250000 cells/well. Experiments were performed with two different donors (*N* = 2) in single measurements or duplicates. Anti‐CD3 monoclonal antibodies (61 ng/ml) were used to activate T cells and OCR and ECAR values were measured for 1 h at 10 consecutive time points. Three groups were compared: An untreated control group (control, *n* = 20), a group pre‐incubated for 2 h with anti‐CD3 (anti‐CD3, *n* = 20), and a third group preincubated with anti‐CD3 and treated with 100 nM copanlisib (anti‐CD3 + copanlisib, *n* = 40). The energy map (Figure 2A) indicates increased glycolysis upon activation of T cells and suppressed glycolysis by copanlisib. This snapshot was confirmed by consecutive analysis presented as Cumming plots (Figure 2B) which showed significantly increased ECAR rates upon activation (control: 39.70 ± 23.92, anti‐CD3: 58.24 ± 29.06) and significantly decreased ECAR rates in the presence of copanlisib (43.16 ± 20.23, ANOVA, *p* < .000). In contrast, OCR rates were only slightly affected, corroborating the assumption that copanlisib specifically impairs glycolysis.

### 3.3 Copanlisib suppresses activation CD4^+^ T cells activation

**Figure 3:**
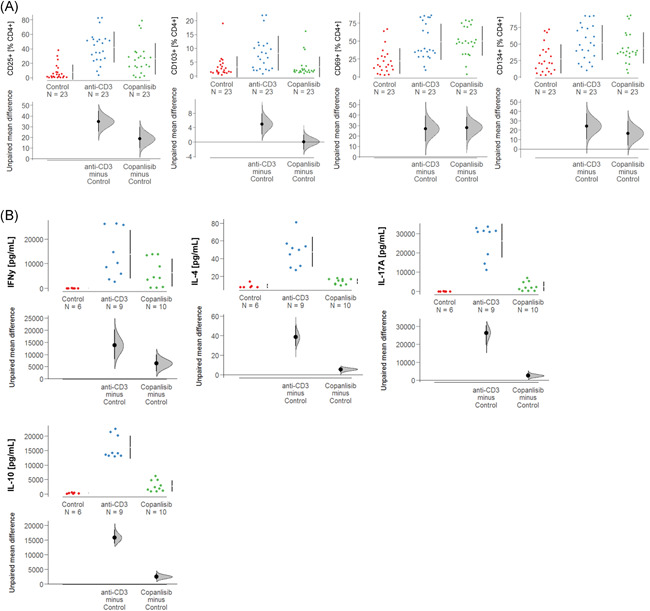
Copanlisib impairs the activation of T cells and secretion of cytokines. PBMCs were incubated in the presence or absence of anti‐CD3 antibodies (62 ng/ml) and copanlisib (100 nM) for 72 h. The experiments were performed on different days. Groups compared: untreated control (control), activated CD4^+^ T cells (anti‐CD3) and activated CD4^+^ T cells + copanlisib (anti‐CD3 + copanlisib). (A) Flow cytometric analysis of frequencies of CD4^+^CD25^+^, CD4^+^CD69^+^, CD4^+^CD103^+^, and CD4^+^CD134^+^ T cells depicted as Cumming plots. Experiments were performed with seven donors (*N* = 7) in triplicates or quadruplicates, number of experiments *n* = 23). (B) Levels of cytokines depicted as Cumming plots. Supernatants of cell cultures were analyzed by Luminex assays (control: experiments were repeated with two donors (*N* = 2, and performed in triplicates, number of experiments *n* = 6; anti‐CD3 and copanlisib: number of donors *N* = 3, experiments were performed in triplicates or quadruplicate, number of experiments *n* = 9, *n* = 10 respectively). The upper part of the plot presents each data point in a swarm plot. The mean and *SD* of each group is plotted as a gapped line, the vertical lines correspond to the mean ± *SD* and the mean itself is depicted as a gap in the line. For the comparison of the groups. In the lower panel of the plots, the effect sizes are shown. The 0 point of the difference axis is based on the mean of the reference group (control). The dots show the difference between groups (effect size/mean difference). The shaded curve shows the entire distribution of excepted sampling. PBMC, peripheral blood mononuclear cell

To analyse the impact of the copanlisib treatment on the activation of T cells, PBMCs were isolated donors and 1 × 10^6^ cells were incubated in the absence or presence of anti‐CD3 antibodies (61 ng/ml) and copanlisib (100 nM) for 72 h as described in Section 2. Cells were subjected to flow cytometric analysis as described in Section 2 for frequencies of the T cell activation markers CD25, CD69, CD134, and CD103 (for gating strategy see Figure S1). Measurements were performed in triplicates or in quadruplicates with PBMC from seven donors (*N* = 7). Three groups were compared: untreated cells (control: number of samples *n* = 23), activated T cells (anti‐CD3, number of samples *n* = 23) and activated T cells treated with copanlisib (copanlisib, number of samples *n* = 23). As shown in Figure 3A exposure to anti CD3 antibodies resulted in significantly increased frequencies of CD4^+^ T cells expressing CD25, CD103, CD134, and CD69 (ANOVA followed by TukeyHSD: CD4^+^ CD25^+^: 42.15 ± 21.46, mean ±* SD, p* = .000; CD4^+^ CD69^+^: 48.97 ± 25.24, *p* = .000; CD4^+^ CD103^+^: 8.21 ± 6.32, *p* = .002, CD4^+^ CD134^+^: 51.87 ± 26.5, *p* = .003) indicating a general activation of CD4^+^ T cells. Copanlisib significantly impaired the activation of CD25^+^ (26.06 ± 21.82, *p* = .013) and CD103^+^ (3.29 ± 3.70, *p* = .002) T cells whereas frequencies of CD69^+^ (50. 05 ± 20.66) and CD134 (44.02 ± 23.68) were unaffected.

As activation of T cells by anti‐CD3 antibodies is known to result in secretion of ILs,^32^ the effect of copanlisib on cytokine levels was examined in supernatants of the cell cultures by Luminex analysis (control: number of donors *N* = 2, number of samples *n* = 6; anti‐CD3: number of donors *N* = 3, number of samples *n* = 9; copanlisib; number of donors *N* = 3, number of samples *n* = 10). As shown in Figure 3B, activation of T cells with anti‐CD3 antibodies resulted in significantly increased expression of IFNγ (control: anti‐CD3: 15139.0 ± 10263.12 pmol/ml, mean ± *SD, p* < .000), IL‐4 (49.20 ± 16.67 pmol/ml, *p* < .000), IL‐17 (26733.0 ± 8387.89 pmol/ml, *p* < .000) and IL‐10 (16002.0 ± 3773.3 pmol/ml, *p* < .000), corroborating the general activation by anti‐CD3 observed in the previous experiment. As expected, copanlisib significantly suppressed the secretion of all examined cytokines of IFNγ (6332.0 ± 5707.61 pmol/ml, *p* = .018), IL‐4 (14.35 ± 3.04 pmol/ml, *p* < .000), IL‐17 (2529.9 ± 2392.19 pmol/ml, *p* < .000) and IL‐10 (2769.0 ± 1892.36 pmol/ml, *p* < .000).

### 3.4 Copanlisib treatment ameliorates pathological phenotype in vivo

To further validate copanlisib as a potential therapeutic for IBD, NSG‐UC mice were treated with copanlisib in two different experiments. Mice were reconstituted with PBMCs from two different UC patients in relapse who were currently treated with vedolizumab and tofacitinib or therapeutically naïve (for patient characteristics and groups of mice, see Table S3). NSG‐UC mice were challenged according to a standard protocol as described in Material and Methods. Seven days post reconstitution the mice were divided into three groups, an unchallenged control group (control, *n* = 8), a group challenged with ethanol and treated intraperitoneally with the carrier methylcellulose (EtOH, *n* = 14) and an ethanol‐challenged group treated with 6 mg/kg copanlisib in methylcellulose (copanlisib, *n* = 14). Except for the unchallenged control mice, all mice were rectally challenged with 10% ethanol on Day 7, followed by challenge with 50% ethanol on Day 14. Copanlisib in 100 µl methylcellulose and 100 µl methylcellulose alone were injected intraperitoneally on Days 7, 8, 14, 15, and 16 into the respective groups.

**Figure 4:**
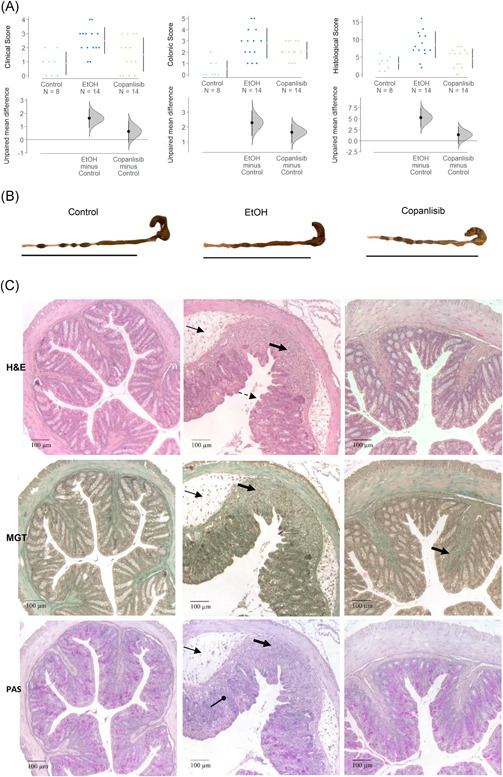
Copanlisib ameliorates symptoms and pathological phenotype in NSG‐UC mice. NSG‐UC mice were engrafted with PBMCs derived from two UC patients, challenged with 10% ethanol at Day 7, and 50% ethanol on Day 14 and treated with 6 mg/kg copanlisib or carrier on Days 6, 7, and 14–16. Groups compared: unchallenged control (control, number of donors *N* = 1, number of mice *n* = 8), ethanol challenged control (EtOH, number of donors *N* = 2, number of mice *n* = 14), ethanol challenged and treated with copanlisib (copanlisib, numer of number of donors *N* = 2, number of mice, *n* = 14). (A) Clinical‐, colon‐ and histological scores depicted as Cumming plots. The upper part of the plot presents each data point in a swarm plot. The mean and *SD* of each group is plotted as a gapped line, where the vertical lines correspond to the mean ± *SD* and the mean itself is depicted as a gap in the line. In the lower panel of the plots, the effect sizes are shown. The 0 point of the difference axis is based on the mean of the reference group (control). The dots show the difference between groups (effect size/mean difference). The shaded curve shows the entire distribution of excepted sampling error for the difference between the means (the higher the peak, the smaller the error). The error bar in the filled circles indicates the 95% confidence interval (bootstrapped) for the difference between means. (B) Representative macrophotographs of colons. (C) Histological manifestations. (a) Hematoxilin (HE), (b) Masson Goldner Trichrome (MGT), (c) Periodic Acid Schiff (PAS). Solid arrows indicate edema and influx of inflammatory cells, dashed arrows epithelial erosions, bold arrows show fibrosis, and arrows with a circle arrowhead indicate goblet cell loss. PBMC, peripheral blood mononuclear cell; UC, Ulcerative colitis

As previously observed,^23^ untreated NSG‐UC mice challenged with EtOH experienced mild weight loss and in some cases, diarrhea, as opposed to unchallenged control mice which appeared normal. Clinical symptoms were classified according to a clinical score as described in Section 2. As shown in Figure 4A, the clinical symptoms increased significantly in ethanol‐challenged mice as compared to nonchallenged mice (control: 0.88 ± 0.84; mean ±* SD*; ethanol: 2.5 ± 0.94; ANOVA followed by TukeyHSD, *p* = .006) and the score slightly improved in response to treatment with copanlisib, albeit not significantly (1.5 ± 1.23, ethanol vs. copanlisib, *p* = n.s.). Macroscopic inspection of the colon corroborated the clinical score. In contrast to colon of control mice, colons of ethanol‐challenged mice exhibited unformed pellets, dilatation and colon shortening. Copanlisib‐treated mice slightly benefitted from treatment (Figure 4B). Appearance of the colons were classified according to a colonic score as described in Section 2. As shown in Figure 4A, the colon score of copanlisib‐treated mice decreased, albeit not significantly (control: 0.5 ± 0.75; ethanol: 2.79 ± 1.37, ANOVA followd by TukeyHSD EtOH vs. control, *p* = .001; copanlisib: 2.14 ± 0.77, copanlisib vs. EtOH, *p* = .069). To further examine the effect of copanlisib, the histopathological manifestations were examined by three stainings: HE stain to colon architecture, MGT for connective tissue (green), and PAS stain to visualize goblet cells.^33^, ^34^, ^35^ As shown in Figure 4C, inflammation in the colon of ethanol‐challenged mice were characterized by edema, influx of a mixed infiltrate into the mucosa and submucosa, epithelial erosions, loss of goblet cells, altered crypt architecture and fibrosis. Treatment with copanlisib reversed the appearance and with the exception of ongoing fibrosis, resulted in an almost normal phenotype. The histological manifestations were classified according to a histological score as described in Section 2. As shown in Figure 4A, the score significantly increased upon challenge with EtOH (control: 3.25 ± 1.83; ethanol: 8.5 ± 3.81, *p* = .001) and significantly decreased in response to treatment with copanlisib (copanlisib: 4.57 ± 2.82, EtOH vs. copanlisb, *p* = .006.).

**Figure 5:**
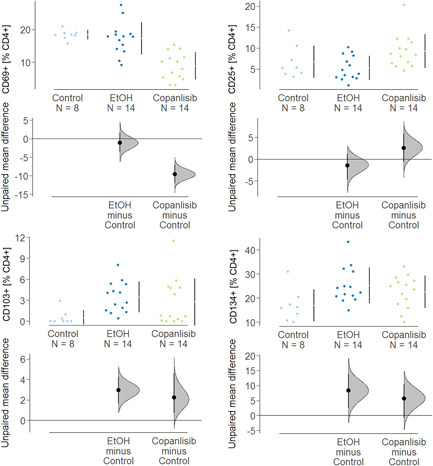
Copanlisisib affects frequencies of early activated CD4^+^ T cells in vivo. Mice were treated as described in Figure 4. Flow cytometric analysis of frequencies of CD4^+^CD69^+^, CD4^+^CD25^+^, CD4^+^CD103^+^, and CD4^+^CD134^+^ T cells depicted as Cumming plots. The upper part of the plot presents each data point in a swarm plot. The mean and *SD* of each group is plotted as a gapped line, the vertical lines correspond to the mean ± *SD* and the mean itself is depicted as a gap in the line. For the comparison of the groups. In the lower panel of the plots, the effect sizes are shown. The 0 point of the difference axis is based on the mean of the reference group (control). The dots show the difference between groups (effect size/mean difference). The shaded curve shows the entire distribution of excepted sampling

To examine the effect of copanlisib on activation of T cells, human leukocytes were isolated from spleen and subjected to flow cytometric analysis. As shown in Figure 5, frequencies of CD4^+^ CD25^+^, CD4^+^ CD69^+^, CD4^+^ CD103, and CD4^+^ CD134^+^ T cells of unchallenged mice were in a similar range as unchallenged PBMC in vitro (Figure 3). Unlike in the in vitro experiment, however, challenge with ethanol solely affected frequencies of CD4^+^ CD103^+^ (control: 0.53 ± 1.02; ethanol: 3.46 ± 2.19, ANOVA followed by TukeyHSD, *p* = n.s.) and CD4^+^ CD134^+^ (control: 16.92 ± 6.66, mean ± *SD*; ethanol: 25.23 ± 6.73, *p* = .04 whereas frequencies of CD4^+^ CD69^+^ and CD4^+^ CD25^+^ remained unaffected. Treatment with copanlisib significantly decreased CD4^+^ CD69^+^ T cells (copanlisib: 8.86 ± 4.17, ethanol vs. copanlisib, *p* < .000), whereas frequencies of CD4^+^ CD25 cells even increased albeit not significantly. This observation is in contrast to the results obtained in vitro where frequencies of CD4^+^ CD25^+^ T cells diminished upon exposure to copanlisib and CD4^+^ CD69^+^ cells remained unaffected.

### 3.5 oPLS‐DA analysis to evaluate the in vivo efficacy of copanlisib

To evaluate the overall efficacy of copanlisib treatment, an orthogonal partial least square analysis (oPLS‐DA) was performed using the clinical‐, colon‐ and histological scores and frequencies of CD4^+^ CD69^+^ and CD4^+^ CD134^+^ T cells as variables. In this analysis, each data point represents one mouse, combining values of the selected variables.

**Figure 6:**
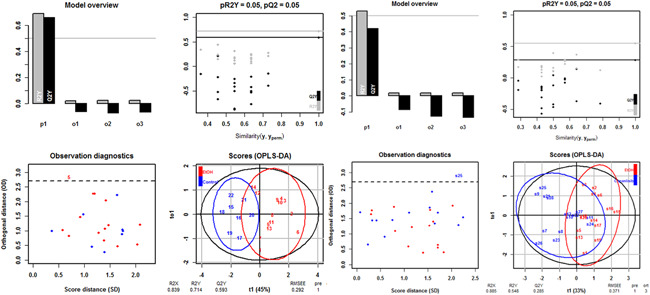
oPLS‐DA analysis support in vivo efficacy of copanlisib. Mice were treated as described in Figure 4. Clinical, colonic, and histological scores and frequencies of CD4^+^ CD69^+^ and CD4^+^ CD134^+^ cells were selected as variables. Orthogonal partial least square discrimination analysis (o‐PLS‐DA). Top left: inertia barplot; Top right: significance diagnostic: the pR2Y and pQ2 of the model are compared with the corresponding values obtained after random permutation of the y response. Bottom left: Outlier diagnostics. Bottom right: X‐score plot: the number of components and the cumulative R2X, R2Y, and Q2Y are indicated below the plot. R2Y: fraction of the variation of the X variables explained by the model, R2X: fraction of the variation of the Y variables explained by the model. Q2Y: fraction of the variation of the Y variables predicted by the model, root mean square error of estimation (RMSEE) value

As depicted in Figure 6

oPLS‐DA revealed a clear distinction between the control versus the ethanol challenged group and ethanol challenged group versus copanlisib treated group. The values of the fraction of the variation of the X variables explained by the model (R2X), of the fraction of the variation of the Y variables explained by the model (R2Y) and of the fraction of the variation of the Y variables predicted by the model (Q2Y) were high in both comparisons, indicating a good fit of the model on the given data. For significance diagnostics, the pR2Y and pQ2 values were compared with values after random permutation of the y response. The obtained values of *p* = .05 corroborated the validity of the oPLS‐model and indicate that both separations from the ethanol‐challenged group were significant.

The separation of the control group from the ethanol challenged group was significant as indicated by the R2X‐value of 0.839, the R2Y‐value of 0.714, the Q2R‐value of 0.53 and the low root mean square error of estimation [RMSEE] value of 0.292). The lower R2X, R2Y, and Q2Y values (0.855, 0.548, and 0.285, respectively) and the higher RMSEE value (0.371) suggested, however, a less pronounced distinction between the copanlisib and the ethanol‐challenged group.

## 4 DISCUSSION

The presentation of antigen to TCR evokes a dual response by activating Na^+^ channels and the PI3K pathway. Activated Na^+^ channels allow for the influx of Ca^++^ into the cells, which in turn activates the transcription factor nuclear factor of activated T cells ultimately resulting in the expression of proinflammatory cytokines.^32^ In UC, this pathway is therapeutically targeted by the calcineurin inhibitor tacrolimus. The PI3K pathway, on the other hand, relays its signal to multiple pathways to include RHO GTPases and the NFκB‐, SH1P‐ and AKT pathways and results in migration, inflammation and proliferation of cells.^13^, ^14^ In this study, the PI3K/AKT pathway was examined as a potential therapeutic intervention point for IBD. The involvement of this pathway in IBD implies a metabolic switch to glycolysis mediated by AKT. Indeed, previous studies examining the metabolic status by mass spectrometry of metabolites in plasma of IBD patients in relapse as compared to patients in remission suggested a switch to glycolysis during acute inflammation.^15^, ^16^ In this study, we confirmed a preference of glycolysis in IBD patients by determining the OCR and ECAR levels by Seahorse analysis. Correlation analysis of both rates indicated a coupling of glycolysis to oxygen consumption in IBD patients that was not observed in non‐IBD individuals. Thus, these results corroborated the observation of the previous study. As we would expect increased glycolysis to be reflected in elevated frequencies of activated T cells in IBD patients, levels of the early activation marker CD69 and the late activation marker CD134 were determined in IBD patients as compared to non‐IBD patients. Both markers were significantly elevated in IBD patients. Thus, this result further corroborated PI3K as a potential therapeutic intervention point.

To validate the PI3K inhibitor copanlisib as a potential therapeutic, the ability to suppress T cell activation was examined in vitro. In vitro, T cell activation by antigen‐presenting cells is usually mimicked by antibodies binding to the T cell coreceptor CD3. We have shown in this study, that anti‐CD3 antibodies activate T cells to express CD4^+^ T cell activation markers and cytokines and that the inhibition of the PI3K pathway by copanlisib specifically suppressed activation represented by CD25^+^ and CD103^+^, but not that of the early or late activation markers CD69 or CD134. CD25^+^ (IL‐2 receptor) is expressed in a huge number of cell types, including CD4^+^ T cells.^36^ The IL‐2R conveys the signal of the pleiotropic cytokine IL‐2 that ultimately promotes proliferation and differentiation.

CD103 has first been identified as a surface marker of mucosal regulatory CD4^+^ T cells.^37^ CD103 stands for integrin αE, which can form a heterodimeric integrin with ß7 on T cells.^37^ Integrin αE/ß7 binds to E‐cadherin expressed on epithelial cells such as intestinal epithelia and thus ensures the residence of these cells in the mucosa and lamina propria.^37^ The αE/ß7 integrin is important in modulating homeostasis of memory T cells by mediating selective retention of these T lymphocytes in the intestinal epithelia and lamina propria through its binding to E‐cadherin.^38^ Therefore, CD103 has become a marker of tissue resident memory T cells, which are now thought to ensure a swift response to an inflammatory assault.^37^ It is interesting that activation of T cells results in a prompt response to direct T cells to the mucosa and that copanlisib may inhibit this response.

CD4^+^ T cells expressing the C‐type lectin receptor CD69 serve as markers for early immune cell activation and tissue retention.^38^, ^39^ Upon TCR/CD3 engagement, CD69 expression is rapidly induced on the surface of T lymphocytes, activating cytokines and mitogenic stimulation.^40^

Finally, CD134 (OX40) is a member of the TNF receptor family. CD134 is considered a late activation marker as it is expressed up to 5 days after activation.^41^, ^42^ CD134 ligate with OX40L, which is expressed on APCs like monocytes, B cells, or dendritic cells.^43^ The ligation of receptor and ligand leads to the formation of signalosomes in membrane lipid microdomains (TNF receptor associated factor), which in turn mediate the interaction with PI3K/AKT and NFκB pathway. Thus, copanlisib may act downstream of CD134 and may not inhibit the expression of CD134.

To further validate copanlisib as a potential therapeutic, it was tested in the NSG‐UC mouse model. Treatment reduced inflammation as indicated by an almost normal macroscopic appearance of the colon and significantly reduced histopathological phenotype. In stark contrast to the results obtained from the in vitro experiments, treatment with copanlisib resulted in significantly reduced frequencies of CD4^+^ T cells expressing the early activation marker CD69 and significantly induced frequencies of CD25^+^ expressing CD4^+^ T cells. These opposing results obtained in vitro and in vivo might be explained by the artificial nature of the CD4 stimulation by anti‐CD3. It is noteworthy that IBD patients express higher levels of CD69^+^ CD4^+^ cells. The suppression of these cells in vivo maybe a further indication that copanlisib may be efficacious in humans. oPLS‐DA analysis validated copanlisib as a potential therapeutic in IBD. However, the fact that complete remission was not achieved by copanlisib suggest that it might be used in combination with other therapeutics such as tacrolimus addressing the alternate T cell activation pathway. The change of metabolism during activation of T cells has also the potential to identify metabolites as biological markers of active disease. This, in combination with an inhibitor of glycolysis could be an important step towards individualized and phase dependent therapies.

## CONFLICT OF INTERESTS

Paula Winkelmann, Anna‐Lena Unterweger, and Diya Khullar are supported by Shaw Research. Roswitha Gropp has a consulting agreement with Shaw Research.

## AUTHOR CONTRIBUTIONS

**Paula Winkelmann, Anna‐Lena Unterweger, and Diya Khullar:** performed the animal studies. **Florian Beigel, Leandra Koletzko:** recruitment of patients, patient history. **Matthias Siebeck:** designed the experiments; **Roswitha Gropp:** writing the manuscript, data analysis, and design of experiments.

## ETHICS STATEMENT

Written, informed consent was given by all donors. The study was approved by the Institutional Review Board (IRB) of the Medical Faculty at the University of Munich (120‐15).

Animal studies were approved by the animal welfare committees of the government of Upper Bavaria, Germany (55.2‐2‐1‐54‐2532‐74‐15) and performed in compliance with German Animal Welfare Laws.

## Supporting information

Supporting information.

Supporting information.

Supporting information.

Supporting information.

## Data Availability

All important data are part of the manuscript.
