# Supplementary material for: The PI3K pathway as a therapeutic intervention point in inflammatory bowel disease
Source: Immun Inflamm Dis. 2021 May 4;9(3):804–18. doi: 10.1002/iid3.435 (PMC8342202; doi:10.1002/iid3.435)
Supplement: Supplementary file 1 — Supporting information. [file IID3-9-804-s004.docx]

**A**

CD4+

CD4+ CD134+

CD4+ CD103+

CD4+ CD69+

CD4+ CD25+

CD4+ RO+ 62L± CCR7±

CD27+ IgD±

**B**

CD4+

CD4+ CD25+

CD4+ CD103+

CD4+ CD69+

CD4+ CD134+

**
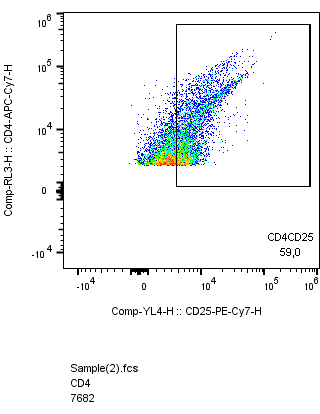
**

**Supplementary Figure 1 Gating Strategy.** (A) Human PBMC; (B) Human leukocytes isolated from mouse spleen;
