# Supplementary material for: The PI3K pathway as a therapeutic intervention point in inflammatory bowel disease
Source: Immun Inflamm Dis. 2021 May 4;9(3):804–18. doi: 10.1002/iid3.435 (PMC8342202; doi:10.1002/iid3.435)
Supplement: Supplementary file 2 — Supporting information. [file IID3-9-804-s001.docx]

**Supplementary Table 1. Antibodies used to label human leukocytes**

| **Surfacemarker** | **Colour** | **Cat # RRID #** |
| --- | --- | --- |
| CD4 | Allophycocyanin (APC)- Cy7 | **BioLegend Cat# 317417, RRID:AB_571946** |
| CD103 | APC | **BioLegend Cat# 350215, RRID:AB_2563906** |
| CD69 | FITC | **BioLegend Cat# 310903, RRID:AB_314838** |
| CD25 | PE/Cy7 | **BioLegend Cat# 302611, RRID:AB_314281** |
| CD134 (Ox40) | PE | **BioLegend Cat# 350003, RRID:AB_10641708** |
