# Supplementary material for: The PI3K pathway as a therapeutic intervention point in inflammatory bowel disease
Source: Immun Inflamm Dis. 2021 May 4;9(3):804–18. doi: 10.1002/iid3.435 (PMC8342202; doi:10.1002/iid3.435)
Supplement: Supplementary file 3 — Supporting information. [file IID3-9-804-s003.docx]

**Table S2 Mean OCR and ECAR mean values and basic patient characteristics**

| **Donor** | **Diagnosis** | **OCR [pmol/min] Mean** | **OCR SD** | **ECAR [mpH/min] Mean** | **ECAR SD** | **SCCAI* / CDAI**** | **Weight** | **Height** | **BMI** | **Age** | **TSD***** | **Medication** |
| --- | --- | --- | --- | --- | --- | --- | --- | --- | --- | --- | --- | --- |
| 1 | CD | 51,98 | 22,80 | 15,80 | 7,60 | 91 | 52 | 1,66 | 18,87 | 33 | 1 | Infliximab, Glucocorticoid |
| 2 | CD | 39,45 | 6,23 | 3,73 | 0,58 | 92 | 66 | 1,72 | 22,31 | 33 | 19 | Infliximab |
| 3 | CD | 60,03 | 18,20 | 13,70 | 3,14 | 141 | 68 | 1,7 | 23,53 | 39 | 19 | Infliximab |
| 4 | CD | 59,09 | 20,41 | 18,70 | 6,44 | 111 | 70 | 1,57 | 28,40 | 57 | 19 | Ustekinumab |
| 5 | CD | 64,44 | 8,32 | 17,43 | 3,70 | 45 | 68 | 1,67 | 24,38 | 34 | 16 | Infliximab |
|  |  |  |  |  |  |  |  |  |  |  |  |  |
| 1 | UC | 23,02 | 8,89 | 4,30 | 3,31 | 3 | 60 | 1,7 | 20,76 | 34 | 14 | Azathioprine, Mesalazine, Golimumab |
| 2 | UC | 83,60 | 8,91 | 25,89 | 5,09 | 0 | 52 | 1,64 | 19,33 | 34 | 6 | Infliximab |
| 3 | UC | 54,92 | 33,07 | 16,42 | 15,17 | 12 | 58 | 1,62 | 22,10 | 24 | 3 | Infliximab, Mesalazine |
| 4 | UC | 73,27 | 21,35 | 27,32 | 4,71 | 4 | 70 | 1,80 | 21,60 | 55 | 15 | none |
| 5 | UC | 84,70 | 15,02 | 30,05 | 4,95 | 2 | 70 | 1,72 | 24,10 | 81 | 50 | Vedolizumab, Glucocorticoid |
|  |  |  |  |  |  |  |  |  |  |  |  |  |
| 1 | non-IBD | 68,37 | 33,73 | 12,24 | 4,13 |  | 74 | 1,83 | 22,10 | 63 |  |  |
| 2 | non-IBD | 86,60 | 29,15 | 18,18 | 1,36 |  | 72 | 1,63 | 27,10 | 52 |  |  |
| 3 | non-IBD | 59,80 | 25,08 | 10,30 | 6,31 |  | 80 | 1,85 | 23,37 | 20 |  |  |
| 4 | non-IBD | 59,14 | 21,34 | 11,67 | 7,41 |  | 90 | 1,7 | 31,14 | 43 |  |  |
| 5 | non-IBD | 57,27 | 29,13 | 24,09 | 7,52 |  | 81 | 1,89 | 22,68 | 23 |  |  |
| 6 | non-IBD | 41,69 | 32,54 | 17,59 | 6,80 |  | 55 | 1,68 | 19,49 | 23 |  |  |

*SCCAI simple clinical colitis activity index

**CDAI Crohn’s disease activity index

***Time since diagnosis
