# Supplementary material for: The PI3K pathway as a therapeutic intervention point in inflammatory bowel disease
Source: Immun Inflamm Dis. 2021 May 4;9(3):804–18. doi: 10.1002/iid3.435 (PMC8342202; doi:10.1002/iid3.435)
Supplement: Supplementary file 4 — Supporting information. [file IID3-9-804-s002.docx]

**Table S3 Patient scores, medication and groups of mice defined post reconstitution**

|  | | | **Groups of mice** | | |
| --- | --- | --- | --- | --- | --- |
| **Donor** | **Medication** | **SCCAI Score** | **Control** | **Ethanol** | **Copanlisib** |
| A | Entivio Tofacitinib | 12 | 4 male 4 female | 4 male 4 female | 4 male 4 female |
| B | none | 7 | - | 4 male 2 female | 4 male 2 female |
